# Implementation of Video Feedback Within a Community Based Naturalistic Developmental Behavioral Intervention Program for Toddlers With ASD: Pilot Study

**DOI:** 10.3389/fpsyt.2021.763367

**Published:** 2021-12-02

**Authors:** Claire B. Klein, Deanna M. Swain, Bethany Vibert, Elysha Clark-Whitney, Amy R. Lemelman, Jennifer A. Giordano, Jamie Winter, So Hyun Kim

**Affiliations:** ^1^Center for Autism and the Developing Brain, Weill Cornell Medicine, White Plains, NY, United States; ^2^Autism Center, Child Mind Institute, New York, NY, United States; ^3^NewYork-Presbyterian Hospital, White Plains, NY, United States

**Keywords:** early intervention (EI), parent-mediated intervention, naturalistic developmental behavioral intervention (NDBI), video feedback, community-based services, technology, autism spectrum disorder (ASD)

## Abstract

Video feedback (VF) is an intervention delivery technique that complements naturalistic developmental behavioral interventions (NDBI) and parent-mediated interventions (PMI) by using caregiver-child interaction videos reviewed with a clinician to facilitate behavioral change in caregivers. Although VF has been implemented in PMI with young children with ASD, examinations of feasibility and acceptability, as well as the potential effectiveness of VF in community settings, have been limited. In this pilot randomized control trial (NCT03397719; https://clinicaltrials.gov/ct2/show/NCT03397719), families were randomized into a state-funded Early Intervention (EI) NDBI program or the NDBI program augmented with VF. Results demonstrated high levels of implementation and acceptability of VF augmenting the community-based EI program in caregivers and clinicians. Both groups showed significant improvements after 6 months in social communication symptoms and some areas of developmental and adaptive skills.

**Clinical Trial Registration:**https://clinicaltrials.gov/ct2/show/NCT03397719, identifier: NCT03397719.

## Introduction

In recent years, research has explored the use of Video feedback (VF) to augment Parent Mediated Intervention (PMI) for young children with various developmental delays (1–3). VF interventions typically consist of filmed caregiver-child interactions that the caregiver watches with a clinician who facilitates guided reflection on caregiver and/or child behaviors. Common intervention targets through VF include parental sensitivity to child cues, child behavior, and parent-child attachment. Across varying clinical populations, treatment purposes, and theoretical orientations, the use of VF with caregivers to deliver some or all intervention has been found to enhance caregiver-child interactions and improve caregiver and child behavior (2, 3).

PMI, or intervention delivery by a parent or caregiver, serves as a core component of many comprehensive treatment models for children with autism spectrum disorders (ASD) (4). In PMIs a clinician guides the caregiver (also referred to as “parent coaching”) to use specific skills during interactions with their child to increase their child's developmental skills and improve the caregiver-child relationship. The inclusion of caregivers in treatment through PMI focuses on the generalization of child skills outside of the clinic setting, with goals to increase caregiver skills or competence and enhance engagement in the caregiver-child dyad (5). PMI has strong empirical support (6), classifying it as an evidence-based practice (5). In fact, many research review panels recommend PMI as an essential feature of early intervention and treatment for individuals with ASD [Autism Intervention Research—Behavioral Network [AIR-B] (7); National Standards Project [NSP] (8)]. In particular, studies suggest that PMI holds the potential for generalization and maintenance of treatment gains that surpass those of intervention delivered directly to the child by a clinician, allowing the caregiver to support the child's development across a wide range of settings (9, 10). Additionally, coaching caregivers based on collaboratively chosen goals can reduce caregiver stress and improve family functioning as a whole (11).

The inclusion of caregivers in treatment is a part of a broader shift toward Naturalistic Developmental Behavioral Interventions (NDBI) for young children with ASD (12). NDBIs are evidence-based treatment approaches for young children with ASD founded in developmental and behavioral learning principles (12). Given the strong support for the use of PMI in treatment for ASD, clinicians have used *in-vivo* coaching to train caregivers to utilize various NDBI strategies, such as providing natural learning opportunities, following the child's lead during interactions, and balancing their role as a social partner (12). In a recent meta-analysis of different types of early intervention, NDBIs stood out as effective (13). NDBIs incorporating PMI have led to gains in social communication, receptive language, joint engagement, play skills, adaptive skills, and cognitive levels (14–19).

VF can be integrated into PMI to maximize the caregiver engagement and learning of various NDBI strategies in the treatment process [see Aldred et al. (1)]. VF used with caregivers of children with ASD to enhance the intervention delivery has been found to improve child language outcomes (20), increase parental self-efficacy (21), increase parental synchrony (22), lead to a long-term reduction in autism symptoms (9), and reduce parental intrusiveness (21). VF is not only clinically useful, but has been well-received by families. Specifically, caregivers have reported strong positive feelings toward VF, indicating that it has allowed them to reflect on their own behavior, learn about their child's behavior, and understand how to implement intervention techniques (23).

Community-based EI services for toddlers with ASD or developmental delays are mandated by the Individuals with Disabilities Education Act (24) and are suggested to be delivered in naturalistic settings. However, not all EI programs provide home-based services, but services may be more center- or classroom-based (25). Thus, interventions such as VF that can be incorporated into everyday activities and routines have been identified as preferable to families of young children with ASD (26). However, despite promising results from past studies on the use of VF in PMI for children with ASD, investigations on the feasibility, acceptability, and potential effectiveness of VF implemented in community-based early intervention (EI) settings have been extremely limited. A handful of studies show the feasibility of VF integrated into community-based interventions, mainly for preschool and school-age children with ASD (27, 28). Studies have also yet to examine the effectiveness of VF in toddlers with ASD, an especially critical developmental period given the downward trend in the age of diagnosis for ASD and the importance of early intervention (29).

In the present study, we conducted a preliminary RCT to examine how VF can augment PMI as a part of a community-based NDBI EI program. Stakeholder input (e.g., clinicians and caregivers) was continuously monitored and incorporated, following participatory research guidelines for adapting evidence-based practice for young children with ASD to community settings (30, 31). Engagement of stakeholders is also critical to maximize program sustainability to maintain fidelity of the intervention when moving from controlled research settings to more natural applications where fidelity may be variable (2, 32). Specifically, we aimed to (1) demonstrate the feasibility of integrating VF within PMI sessions; (2) explore the acceptability of VF from caregivers and clinicians; and (3) compare preliminary treatment effects between the NDBI treatment with and without augmented VF.

## Methods

### Participants

Participants included individuals at the consumer level (i.e., children and caregivers) and service level (i.e., clinicians). Fifteen toddlers with ASD and their caregivers were drawn from a 6-month, community-based, state-funded NDBI EI program which enrolls up to 12 children per year. All children enrolled in the EI program were invited to participate in the study and were randomized into the NDBI vs. NDBI+VF group (see section Procedure). For the NDBI+VF group, the usual caregiver coaching sessions were augmented by VF. There were no significant differences between treatment groups at baseline regarding child and caregiver demographic characteristics as well as hours services received outside of the CADB EI program (Table 1).

**Table 1:** Baseline demographics and tests of group differences (Kruskal–Wallis or Fisher's exact test).

	**NDBI+VF group (*n* = 8)**	**NDBI group (*n* = 7)**	**Whole group (*n* = 15)**	Kruskal–Wallis or Fisher's exact test^*^
	**M (SD) or *n* (%)**	**M (SD) or *n* (%)**	**M (SD) or *n* (%)**	**H or *p***
**Child measures (*****n*** **=** **15)**				
Age (months)	25.63 (5.11)	28.23 (5.29)	26.84 (5.18)	0.97
Sex (males)	5 (62.5%)	5 (71.43%)	10 (66.67%)	1.00
Race				0.65
White	6 (75%)	3 (42.86%)	9 (60%)	
Asian	1 (12.5%)	2 (28.57%)	3 (20%)	
Other	1 (12.5%)	2 (28.57%)	3 (20%)	
**Autism symptom severity**				
*ADOS-2 CSS*				
CSS SA	6.63 (1.77)	7.86 (1.07)	7.20 (1.57)	3.21
CSS RRB	7.25 (3.11)	7.71 (1.5)	7.47 (2.42)	0.01
*BOSCC-clinician*				
SC	26.00 (9.27)	24.93 (5.81)	25.46 (7.46)	0.07
RRB	9.00 (2.58)	8.36 (4.04)	8.68 (3.27)	0.15
*BOSCC-caregiver*				
SC	26.86 (7.56)	21.64 (5.67)	24.25 (6.97)	2.16
RRB	11.00 (3.55)	7.79 (3.25)	9.39 (3.67)	2.77
**Developmental Levels**				
*MSEL or DAS (n = 14; 1)*				
Non-verbal ratio IQ	86.78 (16.92)	80.83 (21.81)	84.00 (18.88)	0.48
Verbal ratio IQ	65.57 (25.41)	65.40 (28.31)	65.49 (25.81)	0.12
*MSEL (n = 14)*				
Visual reception AE	22.50 (7.54)	19.50 (4.59)	21.21 (6.41)	0.71
Fine motor AE	19.38 (3.02)	20.17 (1.94)	19.71 (2.56)	0.02
Receptive language AE	15.50 (7.48)	15.50 (5.75)	15.50 (6.55)	0.01
Expressive language AE	15.38 (5.26)	14.00 (3.74)	14.79 (4.56)	0.04
*Adaptive skills: VABS*				
Communication AE	16.06 (10.75)	17.14 (4.19)	16.57 (8.1)	0.86
Daily living AE	19.00 (8.02)	16.00 (3.65)	17.60 (6.34)	0.41
Motor skills AE	22.75 (7.88)	20.93 (4.31)	21.90 (6.32)	0.03
Socialization AE	13.75 (12.75)	11.29 (4.39)	12.60 (9.55)	0.12
**Hours of services per week (*****n*** **=** **14)**	16.07 (7.18)	10.24 (8.07)	13.16 (7.94)	1.80
**Caregiver measures (*****n*** **=** **15)**				
Age (Years)	41.52 (5.56)	37.04 (4.09)	39.43 (5.29)	1.93
Sex (females)	7 (87.5%)	7 (100%)	14 (93.33%)	1.00
Race				0.15
White	7 (87.5%)	3 (42.86%)	10 (66.67%)	
Asian	1 (12.5%)	3 (42.86%)	4 (26.67%)	
Other	0 (0%)	1 (14.29%)	1 (6.67%)	
Education				0.67
BA/BS or above	7 (87.5%)	6 (85.71%)	13 (86.67%)	
Below BA/BS	1 (12.5%)	1 (14.29%)	2 (13.33%)	
Income				1.00
Below $35,000	1 (12.5%)	0 (0%)	1 (6.67%)	
$81,000–$100,000	2 (25%)	1 (14.29%)	3 (20%)	
$101,000–$130,000	1 (12.5%)	2 (28.57%)	3 (20%)	
Over $161,000	4 (50%)	4 (57.14%)	8 (53.33%)	
Relationship to child				1.00
Mother	7 (87.5%)	7 (100%)	14 (93.33%)	
Father	1 (12.5%)	0 (0%)	1 (6.67%)	
***MONSI-CC (n** **=** **14)***				
Environmental set-up	7.43 (2.35)	9.00 (1.61)	8.21 (2.1)	2.38
Child guided interactions	17.00 (1.87)	18.79 (1.6)	17.89 (1.91)	3.51
Active teaching and learning	25.14 (5.03)	27.21 (2.98)	26.18 (4.12)	0.26
Opportunities for engagement	2.57 (0.98)	3.50 (1.56)	3.04 (1.34)	2.07
Natural reinforcement and scaffolding	13.07 (2.37)	13.71 (1.38)	13.39 (1.89)	0.02
Total score	65.21 (11.26)	72.21 (8.02)	68.71 (10.07)	1.05

* *All values did not reach statistical significance. CSS SA, Comparison score social communication; CSS RRB, Comparison score restricted and repetitive behaviors; BOSCC, Brief Observation of Social Communication Change; MSEL, Mullen Scales of Early Learning; DAS, Differential Ability Scales; AE, age equivalent, VABS, Vineland Adaptive Behavior Scales; MONSI-CC, Measure of NDBI Strategy Implementation—Caregiver Change*.

Clinicians (*n* = 4) were assigned to a family upon entry to the program, prior to randomization. Clinicians included individuals with extensive training in NDBIs including a Psychologist, a Psychologist and Board-Certified Behavior Analyst (BCBA), a Post-Doctoral Fellow in Psychology, and a Speech-Language Pathologist. Two of these clinicians provided care to participants randomized to the NDBI+VF group.

### Procedure

**Figure 1:**
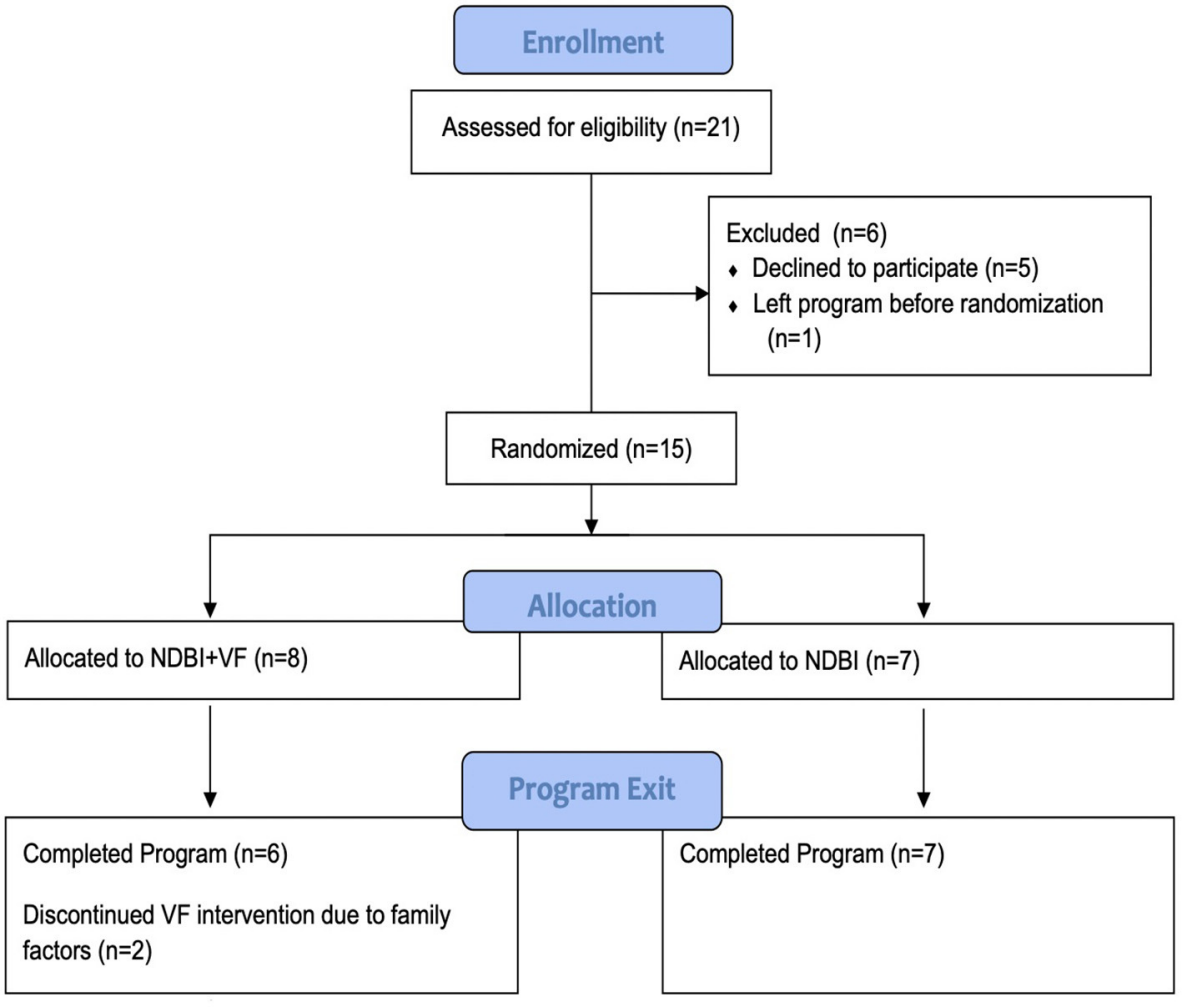
CONSORT diagram.

This study was reviewed and approved by Weill Cornell Medicine Institutional Review Board. After families were assigned a clinician (based on availability), provided their written informed consent to participate in the study, and completed a baseline evaluation, they were randomized into one of two groups, “NDBI” (*n* = 7) vs. “NDBI+VF” (*n* = 8) groups. Randomization was completed by study staff using a concealed allocation sequence (i.e., online random number generator) based on age, gender, and IQ using a matched random assignment process (see CONSORT diagram in Figure 1).

#### NDBI Group

All families received NDBI (standard care), which consisted of group-based (i.e., classroom) clinician-mediated intervention (6 h/week) and individual parent-coaching sessions (3 h/week); see Swain et al. (33) for additional programmatic details. Parent coaching sessions occurred in the center (1 h/week) and also at home (2 h/week) when families lived within 30 min of the center. In addition, children and families received Speech Therapy, Occupational Therapy, and Social Work sessions depending on their Individualized Family Service Plan (IFSP). All caregivers were also invited to participate in weekly 60-min psychoeducation and support groups (2 h/week total). NDBI strategies included but were not limited to: following the child's lead with toy choices; imitating play; sitting face-to-face; providing developmentally appropriate cues; and modeling and prompting for social communication and play (12).

#### NDBI+VF Group

In addition to the NDBI, the NDBI+VF group participated in weekly video-based feedback during the first 10–15 min of one parent coaching session per week. Caregivers and clinicians leveraged novel technological tools (e.g., 360-degree camera and tablet) to prepare for and execute VF sessions. VF centered around the use of NDBI strategies (mentioned above) learned from previous sessions in the recorded home interactions with their child. Clinicians and caregivers collaboratively identified three, 10-min activities and routines that caregivers could carry out with their child at home the week prior to review (30 min total). Caregivers used an LG 360-degree camera to record videos at home without a videographer to ensure naturalistic interactions, allowing the caregiver and child to move freely while remaining in the 360-degree frame. The LG 360-degree cameras did not require the use of the tripod, allowing the camera to be placed on bookshelves, dressers, counters, etc. to capture interactions in the home, or held by hand in the community. These raw videos were then transferred to an iPad by research staff and reviewed by the clinician to prepare for the caregiver coaching sessions. Recordings shorter than 10-min were also included for review by the clinician.

Before each session, clinicians selected two short segments from the caregivers' recordings that week to watch with the caregiver on the iPad, one highlighting an acquired skill for the caregiver (strengths) and one highlighting developing skills (areas for improvement). The length of the segment varied depending on the skill to be highlighted, but was typically around 1–2 min long. To begin the session, clinicians set up preferred toys to keep the child occupied, and began the VF with a video which highlighted a positive attempt from the caregiver or attainment of skill. Next, the clinician asked the caregiver to reflect on the recorded interactions prior to making observations. Then, clinicians noted an antecedent (caregiver action), behavior (child's behavior), and/or consequence (outcome) to highlight as it related to the goals of the family. After providing reinforcement for caregivers' desired behaviors (by pointing out the positive consequences of their behavior), a second video was used to show a future opportunity to utilize new parenting skills. Again, the clinician obtained the caregiver's comments and reflections prior to the clinician making observations. Next, the clinician discussed the skill or technique that would have been helpful during the recorded interaction and supported the caregiver in learning the skills during the current parent coaching session (e.g., modeling, providing handouts, and using examples from the previous or current sessions). Clinicians also allowed the caregiver to ask questions and inquire about the skill. To close the VF component of the session, the clinicians and caregivers worked collaboratively to identify future activities for the caregiver to record in-home.

### Measures

#### Implementation Measures

In order to examine caregiver implementation of VF, the duration of recordings and the number of videos brought in each week were documented. Videos were categorized by type (i.e., play with toys, play without toys, and activities around personal independence and daily living skills). The total dosage of video recording was calculated by dividing the total number of minutes recorded by the number of weeks in the intervention for each caregiver.

Fidelity ratings were assessed regarding clinician treatment implementation of NDBI and VF approaches. Clinicians for both groups were required to meet modified criteria on the ESDM fidelity rating at the beginning of the data collection (34). Clinicians were considered to have met fidelity if they reached no scores under 3 (out of a 1–5 scale) and a mean score of 80% on two consecutively coded joint activity routines. ESDM fidelity was coded by the lead psychologist in the program, an ESDM trainer and an experienced ESDM interventionist (author JW). Fidelity for VF was collected quarterly or when a clinician was assigned to a new child randomized to the VF group. VF fidelity included ratings of pre-planning activities (e.g., the clinician watched the caregiver/child interaction videos prior to the session and noted at least two segments to show the caregiver) and VF session guidelines (e.g., the clinician obtained the caregiver's comments and reflections prior to the clinician making observations). A score of 12 out of 14 (85%) across activities was required to meet fidelity. VF fidelity was completed by raters who achieved inter-rater reliability (85%) across three videos.

#### Acceptability Measures

Caregiver acceptability was measured by attrition rates, service utilization (hours of treatment accessed by each participant), and a Caregiver VF survey. Service utilization was calculated using billing records from classroom and parent coaching sessions. The study-specific Caregiver VF survey was completed anonymously by caregivers in the NDBI+VF group to assess caregiver acceptability after the completion of the program. Survey questions targeted intervention acceptability, practicality, and implementation based on feasibility research guidelines (30).

Clinician acceptability was measured by a VF worksheet used before, during, and after each VF session and a Clinician VF survey given after the completion of treatment for all children in the study. The VF worksheet was used to record notes while preparing videos to review with the caregiver, record caregiver reflections, and included questions such as “How helpful was the video in teaching parent concepts?” and “Did you use the video to inform or direct your coaching during the most recent home session?” (on a 1–10 scale). The Clinician VF survey, completed by two clinicians who provided VF for the study, included open-ended questions to obtain qualitative information regarding programmatic strengths and challenges.

#### Child and Caregiver Outcome Measures

All measures assessing autism symptoms, developmental levels, adaptive functioning, and caregiver strategy use were completed at intervention entry and exit (6 months after entry) by evaluators blind to treatment condition. All the treatment outcome measures used in the study were dimensional which allowed the quantification of changes over time.

##### Autism Symptom Severity

The Brief Observation of Social Communication Change (BOSCC) (35) is a new treatment outcome measure used to quantify changes in social communication skills (SC) and restricted and repetitive behaviors (RRB) in minimally-verbal children based on a 12-min play-based interaction between an adult (e.g., caregiver, clinician) and child. Studies have shown that the BOSCC is more sensitive to changes in core ASD symptoms as compared to the Autism Diagnostic Observation Schedule (ADOS) (35, 36). BOSCC sessions were collected with both caregivers (BOSCC-Caregiver) and clinicians (BOSCC-Clinician) in the clinic to assess improvements in child symptoms across interactants. BOSCC sessions were rated by coders who were blind to treatment condition, time points, and other treatment-related information, and had established reliability. At baseline only, autism symptom severity was measured by the ADOS-2 (37), a semi-structured, standardized, naturalistic assessment. Severity was measured using calibrated severity scores (CSS) for Social Affect (SA) and Restricted and Repetitive Behaviors (RRB) domains (38).

##### Developmental Levels

The Mullen Scales of Early Learning (MSEL) (39) or Differential Abilities Schedule (DAS-II) (40) were used to measure child verbal and non-verbal abilities at entry (n_MSEL_ = 14, n_DAS_ = 1) and exit (n_MSEL_ = 11, n_DAS_ = 1). The MSEL and DAS-II have shown high convergent validity in previous studies of children with ASD (41, 42). Nonverbal and verbal mental ages (NVMA and VMA) were calculated from both measures to examine changes. NVMA was calculated by averaging the age equivalents (AEs) on the Visual Reception and Fine Motor subscales on the MSEL and the Picture Similarities and Pattern Construction subtests on the DAS-Early Years. VMA was calculated by averaging the AEs on the Receptive Language and Expressive Language subscales of the MSEL and the Verbal Comprehension and Naming Vocabulary subtests on the DAS-Early Years. Ratio IQs were derived by dividing nonverbal (NVRIQ) or verbal (VRIQ) mental age by the chronological age in months. NVRIQ and VRIQ were used to quantify baseline IQ scores, while MSEL domain age equivalents (AEs) were used to capture developmental changes over time for consistency (only one child was given the DAS).

##### Adaptive Skills

The Vineland Adaptive Behavior Scales, 2nd and 3rd editions (VABS) (43), a parent interview, was used to measure adaptive functioning. AEs were used to capture changes over time for the Communication, Daily Living Skills, Socialization, and Motor Skills domains.

##### Caregiver NDBI Implementation

The Measure of NDBI Strategy Implementation–Caregiver Change (MONSI-CC) (44) was used to examine changes in caregivers' NDBI strategy use. BOSCC-Caregiver and MONSI-CC ratings were based on the same segments of 12-min caregiver-child interaction videos. The MONSI-CC yields scores in five domains (Environmental Set-up, Child-Guided Interactions, Active Teaching and Learning, Opportunities for Engagement, and Natural Reinforcement and Scaffolding) and a Total Score. Total scores may range from 20 to 100, with higher scores indicating effective and appropriate use of strategies taught in NDBI. The MONSI-CC was rated by coders who had established reliability on the measure and were blind to treatment condition, time points, and other treatment-related information.

### Data Analysis

Analyses were conducted for the 13 children who had completed 6 months (M_NDBI+VF_ = 5.16, SD = 0.98; M_NDBI_ = 4.74, SD = 0.54) of intervention. Caregiver implementation of VF was evaluated using the total dosage of video recordings and video categorization, while clinician implementation of VF was evaluated by VF fidelity ratings. To evaluate the acceptability of NDBI+VF by caregivers, attrition rates were compared between the two groups using Fisher's exact test, an independent samples *t*-test was used to compare mean group differences regarding parent coaching and classroom intervention service utilization, means and SDs were obtained from the Caregiver VF survey. To examine the acceptability of NDBI+VF in clinicians, we also obtained means and SDs from VF worksheets. Additionally, an independent samples *t*-test was used to compare mean differences between families receiving home and clinic sessions vs. clinic sessions only on the VF worksheets.

Given the small sample size in NDBI+VF and NDBI groups, non-parametric statistics were used for analyses to compare the treatment effects between the groups. First, the Kruskal–Wallis test was used to test differences in all outcome measures between the NDBI+VF and NDBI groups at intervention entry. Next, the Wilcoxon signed-rank test was used to examine significant change within each treatment group as well as across both groups for BOSCC-Clinician and Caregiver SC and RRB domain totals, MSEL domain age equivalents, Vineland domain age equivalents, and MONSI-CC domain and total scores. Effect size was calculated using [*r* = *Z*/sqrt(*N*)], with the interpretation of r values as follows:.5 = large effect, .3 = medium effect, .1 = small effect (45, 46). For variables that showed significant change from these analyses in one or both groups, Reliable Change Index (RCI) (47, 48) scores were calculated to examine percentages of participants showing statistically significant change for each treatment group. RCIs were calculated using the formula: $SE_{Diff}=SD_{1}\times \sqrt{2\times \sqrt{1-r}}$ based on the SD of our sample at intervention entry and test–retest reliability from instrument manuals or literature. RCIs were followed up with Fisher's exact tests between the NDBI+VF and NDBI groups to confirm whether there is a significant difference in the proportion of children positive change, no change, or negative change. Spearman's rho non-parametric r correlations were used to examine the association between caregiver (MONSI-CC) and child (BOSCC-Clinician and Caregiver) changes.

## Results

### Caregiver Feasibility

#### Caregiver Implementation

Caregivers in the NDBI+VF group recorded an average of 8.05 total hours of caregiver-child interaction videos (SD = 5.92, Range = 2.46–18.73) across an average of 39.5 videos (SD = 28.53, Range = 14–94) over the course of the 6-month intervention. Each week, caregivers recorded an average of 20.33 min (SD = 14.55, Range = 13.40–46.83) of interactions. Caregivers recorded interactions on average for 60% of weeks during the 6 months of intervention (SD = 16%, Range=33–75%). For each VF session that occurred, the clinician reviewed an average of 33.15 minutes (SD = 15.46) prior to each session.

Across all videos recorded by the caregivers, 63% of videos captured play activity with toys (e.g., play at a table, reading books), 9% captured play without toys (e.g., singing, dancing, playing on a playground), 27% captured activities around personal independence and daily living skills (e.g., feeding, dressing, bath time, and outdoor safety), and 3% were not viewable (e.g., a video was blurry or a file was corrupt).

#### Caregiver Acceptability

Both groups demonstrated acceptable attrition rates (M_NDBI+VF_ = 25% [*n* = 2]; M_NDBI_ = 0%). Results from Fisher's exact test showed that there was no statistically significant association between group and attrition rate (*p* = 0.27).

No group differences in service utilization (hours of treatment by each participant) were found between the NDBI+VF and NDBI groups. Weekly service utilization hours were *M* = 2.16 (SD = 0.58) h for individual parent coaching and M=5.08 (SD=0.46) hours of classroom intervention. Results from an independent samples *t*-test showed that there were no differences between the NDBI+VF and the NDBI groups in the hours of parent coaching [M_NDBI+VF_ = 2.07, SD = 0.53, M_NDBI_ = 2.15, SD = 0.65; *t*_(11)_ = 0.21, *p* = 0.84] or classroom intervention [M_NDBI+VF_ = 5.18, SD = 0.27, M_NDBI_ = 4.97, SD = 0.60; *t*_(11)_ = −0.80, *p* = 0.44].

Results from the Caregiver VF Survey for families in the NDBI+VF group (on a scale of 1–7, 7 being the highest) were available for 4 caregivers (66%). Questions regarding practicality found that caregivers easily operated the camera (M = 7.00, SD = 0.43), understood how to record videos (M = 7.00, SD = 0.00), found time to carry out recordings (M = 5.25, SD = 1.30), incorporated VF into daily routines (M = 5.25, SD = 1.30), and felt that they had enough time with their clinician for VF sessions (M = 6.75, SD = 0.43). Regarding ratings of implementation, caregivers reported that they worked with the clinician to decide what to record (M = 6.75, SD = 0.43) and followed through with the recordings (M = 6.25, SD = 0.83). In regard to acceptability and satisfaction, caregivers reported that watching video in session helped their learning (M = 7.00, SD = 0.00) and they felt that they benefited from parent coaching sessions (M = 6.75, SD = 1.25). Caregivers also rated that the recordings benefited their child (M = 5.00, SD = 1.73), and all caregivers said they would recommend VF to other families (M = 7.00, SD = 0.00). Caregivers reported that they had no recommended changes about the VF component of the intervention. However, difficulties reported by caregivers included constraints on time, concerns about being recorded, having their homes recorded, and distracting the child during the VF session. Caregivers most enjoyed capturing and receiving feedback on their interactions in naturalistic settings. Caregivers also reported that VF helped them to understand themselves and their children better. Caregiver feedback was incorporated through requested modifications to the protocol (e.g., some families requested a text reminder over the weekend to remember to record videos for NDBI+VF; some families requested that they record videos on their own devices when the camera was not readily accessible although this was rare; families requested an individualized approach to homework allowing for the flexibility to choose routines based on their needs).

### Clinician Feasibility

#### Clinician Implementation

The mean fidelity score for VF was 12.5 for 10 sessions (12 of 14 needed to meet fidelity) across the two clinicians that implemented the VF intervention.

#### Clinician Acceptability

Clinician VF worksheet data were obtained from 70 VF sessions across the six children in the NDBI+VF group. Responses to “How helpful was the video in teaching parent concepts” (on a scale of 1–10 with 10 being the highest) had a mean of 7.71 (SD = 1.76). When families were split by those with home sessions (*n* = 4) and those with no home sessions (*n* = 2), clinicians working with families whose sessions were limited to the clinic (parent coaching was *not* delivered in the home) reported VF significantly more helpful (M = 9.09, SD = 0.71) than those working with families whose sessions were held both in the home and in the clinic [M = 6.10, SD = 1.15; *t*_(61)_ = 12.62, *p* < 0.001]. Clinicians reported that 64% of the time, the video informed or directed their most recent parent coaching session. Clinicians also reported that they worked collaboratively with caregivers to select home recording activities for 83% of the sessions.

Of the four clinicians who participated in the study, two clinicians were assigned to children who were randomized to the NDBI+VF group. In response to open-ended questions about what they liked most about VF, clinicians reported that it gave insight into the child's behavior in the home for families without home sessions, and into caregiver-child interactions without the presence of clinicians for families with and without home sessions. Clinicians reported that barriers to VF included the amount of time needed to prepare the session and that it sometimes feels cumbersome for some caregivers to record the recommended amount. One clinician reported that VF sessions often sparked important questions about caregiver techniques that there is not always time to address in the child-focused session, and it may be helpful to have a separate time for the feedback.

### Child and Caregiver Behavior Change

Results from the Kruskal–Wallis test of initial differences showed that there were no significant differences between the NDBI+VF and NDBI groups at intervention entry for all baseline and outcome measures (all *p* > 0.05; Table 1).

#### Autism Symptom Severity

Using Wilcoxon signed-rank tests, the NDBI+VF group showed significant change in SC on the BOSCC-Clinician and Caregiver as well as significant change in BOSCC-Caregiver RRB (Table 2). The NDBI group showed significant change in the BOSCC-Clinician and Caregiver in SC. BOSCC-Clinician SC effect sizes were large for both groups. RCIs revealed that impairments in SC and RRBs measured by the BOSCC scores in the NDBI+VF group decreased in 2 out of 6 (33%) cases for BOSCC-Clinician SC and BOSCC-Caregiver SC and RRB. In the NDBI group, reliable decreases were shown in 2 out of 6 (33%) cases for BOSCC-Clinician SC and BOSCC-Caregiver RRB and no cases for BOSCC-Caregiver SC (Table 3). Based on the Fisher's exact test, the proportion of subjects showing reliable change did not differ by group.

**Table 2:** Wilcoxon signed-ranks test for change from Intervention Entry to Exit.

		**NDBI Group**				**NDBI+VF Group**				**Whole group**			
		***n***	***Z***	***p***	***Effect size r***	***n***	***Z***	***p***	***Effect size r***	***n***	***Z***	***p***	***Effect size r***
**Child measures**													
Autism symptom severity BOSCC-Clinician		6				6				12			
	SC		**−1.997**	**0.05**	**−0.82**		**−2.023**	**0.04**	**−0.83**		**−2.758**	**0.01**	**−0.80**
	RRB		−0.405	0.69	−0.17		−1.153	0.25	−0.47		−1.068	0.29	−0.31
BOSCC-Caregiver		6				6				12			
	SC		**−2.201**	**0.03**	**−0.90**		**−1.992**	**0.05**	**−0.81**		**−2.943**	**0.00**	**−0.85**
	RRB		−1.261	0.21	−0.51		**−2.207**	**0.03**	**−0.90**		**−2.559**	**0.01**	**−0.74**
Developmental Levels: MSEL		5				6				11			
	Visual Reception AE		**−2.032**	**0.04**	**−0.91**		**−2.207**	**0.03**	**−0.90**		**−2.941**	**0.00**	**−0.89**
	Fine Motor AE		−1.483	0.14	−0.66		**−1.997**	**0.05**	**−0.82**		**−2.493**	**0.01**	**−0.75**
	Receptive Language AE		**−2.023**	**0.04**	**−0.90**		−1.782	0.08	−0.73		**−2.669**	**0.01**	**−0.80**
	Expressive Language AE		**−2.023**	**0.04**	**−0.90**		**−1.992**	**0.05**	**−0.81**		**−2.756**	**0.01**	**−0.83**
Adaptive Skills: VABS		7				6				13			
	Communication AE		**−2.371**	**0.02**	**−0.90**		**−2.201**	**0.03**	**−0.90**		**−3.183**	**0.00**	**−0.88**
	Daily Living AE		−1.609	0.11	−0.61		**−2.207**	**0.03**	**−0.90**		**−2.765**	**0.01**	**−0.77**
	Motor Skills AE		**−2.197**	**0.03**	**−0.83**		**−2.032**	**0.04**	**−0.83**		**−2.982**	**0.00**	**−0.83**
	Socialization AE		**−2.201**	**0.03**	**−0.83**		**−1.997**	**0.05**	**−0.82**		**−2.904**	**0.00**	**−0.81**
**Caregiver measures**	
MONSI-CC		6				6				12			
	Environmental Set-Up		−1.782	0.08	−0.73		−1.156	0.25	−0.47		**−1.965**	**0.05**	**−0.57**
	Child Guided Interactions		−1.577	0.12	−0.64		−1.472	0.14	−0.60		**−2.161**	**0.03**	**−0.62**
	Active Teaching and Learning		−1.472	0.14	−0.60		−0.315	0.75	−0.13		−1.337	0.18	−0.39
	Opportunities for Engagement		−1.490	0.14	−0.61		−1.761	0.08	−0.72		**−2.197**	**0.03**	**−0.63**
	Natural Reinforcement and Scaffolding		−0.213	0.83	−0.09		−1.841	0.07	−0.75		−1.132	0.26	−0.33
	Total Score		−1.787	0.07	−0.73		−1.261	0.21	−0.51		**−2.159**	**0.03**	**−0.62**

*BOSCC, Brief Observation of Social Communication Change; MSEL, Mullen Scales of Early Learning; AE, age equivalent, VABS, Vineland Adaptive Behavior Scales, MONSI-CC, Measure of NDBI Strategy Implementation—Caregiver Change. Bolded numbers indicate statistical significance p ≤ 0.05*.

**Table 3:** Reliable Change Indices from Intervention Entry to Exit.

**Measure**	**Difference score for RCI**	**NDBI+VF group**				**NDBI group**			
		***n***	**% RC+**	**% RC0**	**% RC–**	**n**	**% RC+**	**% RC0**	**% RC–**
**BOSCC-clinician**
SC	8.55	6	0%	67%	33%	6	0%	67%	33%
**BOSCC-caregiver**
SC	7.30	6	0%	67%	33%	6	0%	100%	0%
RRB	3.75	6	0%	67%	33%	6	0%	67%	33%
**MSEL AE**
Visual reception	7.95	6	67%	33%	0%	5	40%	60%	0%
Fine motor	3.10	6	67%	33%	0%	5	40%	60%	0%
Receptive language	8.23	6	67%	17%	17%	5	80%	20%	0%
Expressive language	5.93	6	67%	33%	0%	5	60%	40%	0%
**VABS AE**
Communication	6.36	6	83%	17%	0%	7	43%	57%	0%
Daily living	5.83	6	67%	33%	0%	7	57%	43%	0%
Socialization	7.01	6	67%	33%	0%	7	71%	29%	0%
Motor skills	5.54	6	67%	33%	0%	7	43%	57%	0%

*Difference Score for RCI was the amount of change needed between entry and exit to reach statistical significance using the SDs of the sample at intervention entry. BOSCC, Brief Observation of Social Communication Change; MSEL, Mullen Scales of Early Learning; AE, age equivalent; VABS, Vineland Adaptive Behavior Scales*.

#### Developmental Levels

Based on the Wilcoxon signed-rank test, developmental levels measured by the MSEL AE showed significant change in the NDBI+VF group in visual reception, fine motor, and expressive language (Table 2). The NDBI group showed significant change in visual reception, receptive language, and expressive language. Effect sizes were large for all domains for both groups. RCI revealed that there was a reliable increase in 4 out of 6 (67%) of cases across all domains in the NDBI+VF group. The NDBI group showed a reliable increase in 2–4 out of 5 (40–80%) cases across the domains (Table 3). Results from the Fisher's exact test found no significant differences in reliable change between the two groups across all subscales.

#### Adaptive Skills

On the VABS, the Wilcoxon signed-rank test revealed that the NDBI+VF group showed significant change across all domain AEs; the NDBI group showed significant change on all but one domain AEs (i.e., daily living; Table 2). Effect sizes were large for both groups. Based on RCI, 5 out of 6 (83%) cases in the NDBI+VF group showed a reliable increase in communication. Additionally, 4 out of 6 (67%) cases in the NDBI+VF group showed a reliable increase in daily living, socialization, and motor skills. In the NDBI group, 3–5 out of 7 (43–71%) cases showed a reliable increase across domains (Table 3). Based on the Fisher's exact test, there were no significant differences in reliable change between the two groups across all subscales.

#### Caregiver NDBI Implementation

Based on Wilcoxon signed-rank test, the MONSI-CC showed no significant changes in the NDBI+VF or NDBI groups. Effect sizes ranged from small to moderate levels for both groups across different domains. When both groups were combined, significant improvements in NDBI strategies were noted in Environmental Set-Up, Child Guided Interactions, and Opportunities for Engagement (Table 2).

#### Association Between Caregiver and Child Changes

Spearman's rho non-parametric correlations between changes in child BOSCC-Clinician and Caregiver scores and MONSI-CC total score showed that improvement in child social communication symptoms over time measured by the BOSCC-Clinician was significantly correlated with improvement in caregiver implementation of NDBI strategies over time measured by the MONSI-CC Total Score for the NDBI+VF group (*r* = −0.83, *p* = 0.04) but not for the NDBI group (*r* = –0.37, *p* = 0.47).

## Discussion

This pilot RCT examined the implementation, acceptability, and feasibility of VF as an augmentation to PMI NDBI within a community-based EI program for children with ASD. VF was successfully integrated into parent coaching sessions, with clinicians reporting that the intervention was helpful in coaching caregivers. Additionally, caregiver implementation and acceptability measures found caregivers in the NDBI+VF group recorded a sufficient amount of video to facilitate the intervention and no differences in attrition rates or service utilization between the groups. Caregivers reported that VF was beneficial for themselves and their children and helped them to learn NDBI strategies. Preliminary treatment effects between the NDBI+VF and NDBI groups showed comparable amounts of change in social communication symptoms between the groups with varying treatment effects in some developmental and adaptive skills.

### Implementation, Acceptability, and Feasibility of NDBI+VF

This study demonstrated the feasibility of integrating VF into a community-based EI program, from both caregiver and clinician perspectives. Caregiver implementation of VF was acceptable, with most families recording home interactions close to the clinician-recommended dosage of 30-min weekly. All but one caregiver who completed the program agreed that they had time to complete recordings and were able to incorporate the recordings into daily routines. One family reported that they felt limited by the busy schedule due to other commitments such as work and other educational and treatment services. Attrition rate and service utilization were not affected by adding VF to the existing, comprehensive EI (NDBI), suggesting that VF did not add any extra burden to the families and can be successfully integrated into community-based EI. Informal feedback from families who refused to participate in or dropped out of the study revealed a busy schedule and a lack of support system as possible barriers. Caregivers expressed high satisfaction with VF and believed that the VF was beneficial for their children. For caregiver behavioral change to occur in VF, it is important that families buy into the utility of the video recording and review (1), as they did in the present study. Our VF intervention allowed for the inclusion of caregivers not just as passive recipients of intervention, but also in the roles of intervention collaborator and agent of the intervention (49), promoting caregiver buy-in to the intervention. In addition, caregivers in the NDBI+VF group reported increased insight into their own interactive strategy implementation because they reported that VF helped “to understand how we can play with our kids,” and “to find my shortcomings,” consistent with reflections of caregivers who received VF in past studies (23, 27, 50).

Clinician insight regarding the implementation of VF revealed that VF may be especially useful for families without access to home sessions. EI services are sometimes limited to center-based interventions in the U.S., which limits generalizability to naturalistic settings (25). Clinicians reported that VF gave insight into home routines and behavior occurring in the home setting outside of their presence, even for families who could not receive home sessions. In fact, the recording of home interactions also allowed for caregivers to record and clinicians to review the routines that were not always feasible for clinicians to be present for, such as early morning or bedtime routines. Additionally, receiving feedback on behavior in naturalistic settings is believed to aid in the generalization of caregivers' skills learned in the clinic to the home setting (19) and contribute to the utility of the VF intervention (1, 22, 51). While we did not collect data systematically on the reasons why sometimes the videos did not directly inform the coaching session, clinicians anecdotally reported that the focus of the particular session did not always align with the feedback given to the homework videos reviewed that day, although in general, the videos were helpful to inform the overall intervention goals and monitor progress over time.

The incorporation of technology (i.e., 360-degree cameras and tablets) may have also bolstered caregiver and clinician adherence to intervention implementation due to its portable nature and ease of execution. Interventions for children with ASD have increasingly leveraged technological resources, including clinician-mediated parent coaching and behavioral assessment (52). In the current intervention, the availability of small, portable, high-quality cameras allowed for the extension of technological tools into the home environment, without the need for resources such as an additional videographer and with minimal loss of data. This also reduced the efforts of research staff who did not have to make home visits and minimized the effect of an observer on the dyadic interaction. Furthermore, the cameras were provided to families at a relatively low cost to the program. As such, the opportunity to engage families in VF was not dependent upon the family having specific technology in the home, or even internet connection, underscoring the possibility of VF implemented across families with various socioeconomic backgrounds.

Caregiver and clinician surveys revealed barriers to community-based implementation of VF. Feedback from clinicians included the notable amount of time needed to devote to preparing the VF session, highlighting the importance of administrative support as well as the importance of clinician buy-in to see the benefit of the model. As mentioned above, caregiver surveys revealed that the largest barrier may be finding time for recordings in daily routines given other commitments and busy schedules. For some families, it may also be more appropriate to deliver additional VF sessions without the presence of a child, as occupying the child during feedback has been identified as a challenge in previous research as well (50). In this adult-only setting, the session may be devoted to providing feedback with minimal distractions and sufficient time for discussion. However, integrating VF within the *in-vivo* coaching sessions with caregivers and the children, as in the current intervention, provides opportunities to apply the feedback right away during the session. Therefore, the utility of providing a separate VF session may depend on the specific needs of the family. In addition, many of these barriers identified may be even more pronounced in under-resourced families. This highlights the need for future studies with more diverse samples to examine additional barriers to the implementation of VF in various community settings.

### Analyses of Child and Caregiver Changes

The interpretation of the results from the child and caregiver analyses warrants caution given the preliminary nature of the study with a small sample size. In the current pilot RCT, child and caregiver gains were noted across *both conditions*. Children from both groups showed significant improvements in social communication impairments, visual reception, expressive language, as well as adaptive communication, motor, and socialization skills. Caregivers also demonstrated improved use of NDBI strategies. Children in the NDBI+VF group showed significant improvement in fine motor and only marginal improvement in receptive language, whereas receptive language improvement in the NDBI group reached statistical significance. The results may suggest that VF embedded in a comprehensive, community-based NDBI program may not have yielded additional social communication symptom reduction, improvements in developmental levels in young children with ASD, or increased NDBI strategy use for caregivers beyond the gains from the NDBI program alone, although further replications are needed. However, children in the NDBI+VF group demonstrated additional areas of improvement in comparison to those in the NDBI group, including gains in adaptive daily living skills and RRB symptom reduction. This may be reflective of caregivers who received VF having increased opportunities to receive coaching in this area given that nearly a third of videos recorded focused on these skills (e.g., dressing, bath time, and feeding). The improvement in the RRB domain (which includes behaviors such as repetitive play acts, fixated interests) may be explained partly because children's play routines and themes have broadened and become less rigid while interacting with caregivers, which was one of the major targets of VF based on home play interactions. This effect on RRBs following PMI focused on social communication and play has also been found in other NDBI (53). Given the small sample size, future studies with more diverse samples should explore the benefits of VF on daily living and play skills in young children with ASD, in addition to *in-vivo* parent coaching, especially when home-based intervention is not feasible. Finally, for children in the NDBI+VF group, decreases in social communication symptoms with clinicians were significantly associated with improvements in caregivers' use of NDBI strategies. This positive relationship in the NDBI+VF group aligns with previous VF findings that showed increases in caregiver created opportunities (21) and caregiver synchrony as a mediator of child communication outcomes (9).

### Limitations and Future Directions

Caregiver VF surveys were collected from 66% of the NDBI+VF group, and because the surveys were completed anonymously, we could not statistically compare the characteristics of the 4 families that completed the Caregiver VF Survey and those that did not. Therefore, it is important to note that the survey data may not represent the experiences of all families involved, and replications are needed before the results on the acceptability of the VF are generalized. Although the preliminary findings on treatment effects are promising, they should be considered within the context of limitations. For example, because of missing data for certain measures, the direct comparison of results among different instruments is not feasible. In addition, the lack of group differences noted in developmental and adaptive skills may be a result of several factors. Primarily, our intervention was a pilot RCT and featured a small sample size with low power. Furthermore, although measures like the MSEL and VABS are standardized to allow for direct comparison of participants to similarly aged peers, their focus on relatively broad areas of development may preclude their ability to capture hypothesized finer grain change in families receiving the VF component. For example, changes in children's feeding behaviors after using VF sessions to focus on food tolerance may only be captured by a few items in the VABS daily living skills domain, and thus would not be reflected in significant changes in overall scores.

An additional limitation warranting consideration is that the VF dosage recommended in the current study may have been cumbersome for some caregivers, as has been reported in previous VF interventions (28). While many families in the current study recorded the recommended amount of interactions or more, there was variability across families. However, measurement of the factors surrounding caregiver motivation to engage with the intervention to maximize the effectiveness of VF (1) has been difficult to implement (2) and was outside of the scope of the study. Furthermore, there were a few families in the broader EI program who did not want to participate in the study. Although, we were not able to gather information on the reasons why they declined to participate in the study and why some families discontinued the intervention, future research may explore ways to adapt recommendations for dosage of recording based on the family's needs and to identify factors contributing to caregiver motivation to engage in VF and barriers to incorporating VF in daily routines. Additionally, more research is required to fully understand the utility of VF not only in conjunction with *in-vivo* sessions with the children, but also in replacement of them. If VF can be used to provide the appropriate amount of support for some families who have limited access to services, future studies should also examine the validity of VF incorporated into remote, telehealth-based interventions. The cost of the 360-degree cameras and transferring of videos to another device may also be a barrier to the incorporation of VF in community settings or remote interventions, thus future research may consider VF leveraging more readily available technology (i.e., smartphone videos).

### Conclusion

Results from the current preliminary study demonstrate the initial feasibility of VF in a community-based EI program. Caregivers successfully implemented the VF intervention in their daily routines and reported high acceptability toward the intervention. Clinicians delivered VF to fidelity within their intervention sessions and believed VF was an effective tool to teach caregivers NDBI strategies. Findings showed comparable gains in child and caregiver skills, with some additional areas of improvement in children with ASD participating in the NDBI+VF intervention, although the results should be replicated with larger, more diverse samples before they can be generalized into other contexts.

## Data Availability Statement

The raw data supporting the conclusions of this article will be made available by the authors upon request while following the institutional regulations.

## Ethics Statement

The studies involving human participants were reviewed and approved by Weill Cornell Medicine Institutional Review Board. Written informed consent to participate in this study was provided by the participants' legal guardian.

## Author Contributions

CK participated in the design, statistical analysis, interpretation of data, performed measurement, and drafted the manuscript. DS, BV, AL, JG, and JW participated in the data collection and helped to draft the manuscript, and interpretation of the data. EC-W helped to analyze the data and draft the manuscript and performed the measurement. SK conceptualized the study, participated in its design, coordination, interpretation of data, statistical analysis, and drafted the manuscript. All authors read and approved the final manuscript.

## Funding

This work was supported by the Louis and Rachel Rudin Foundation.

## Conflict of Interest

The authors declare that the research was conducted in the absence of any commercial or financial relationships that could be construed as a potential conflict of interest.

## Publisher's Note

All claims expressed in this article are solely those of the authors and do not necessarily represent those of their affiliated organizations, or those of the publisher, the editors and the reviewers. Any product that may be evaluated in this article, or claim that may be made by its manufacturer, is not guaranteed or endorsed by the publisher.
