# Supplementary figures and images for: Implementation of Video Feedback Within a Community Based Naturalistic Developmental Behavioral Intervention Program for Toddlers With ASD: Pilot Study
Source: Front Psychiatry. 2021 Dec 2;12:763367. doi: 10.3389/fpsyt.2021.763367 (PMC8674462; doi:10.3389/fpsyt.2021.763367)

**Supplementary Figure 1.** Reliable Change Graphs


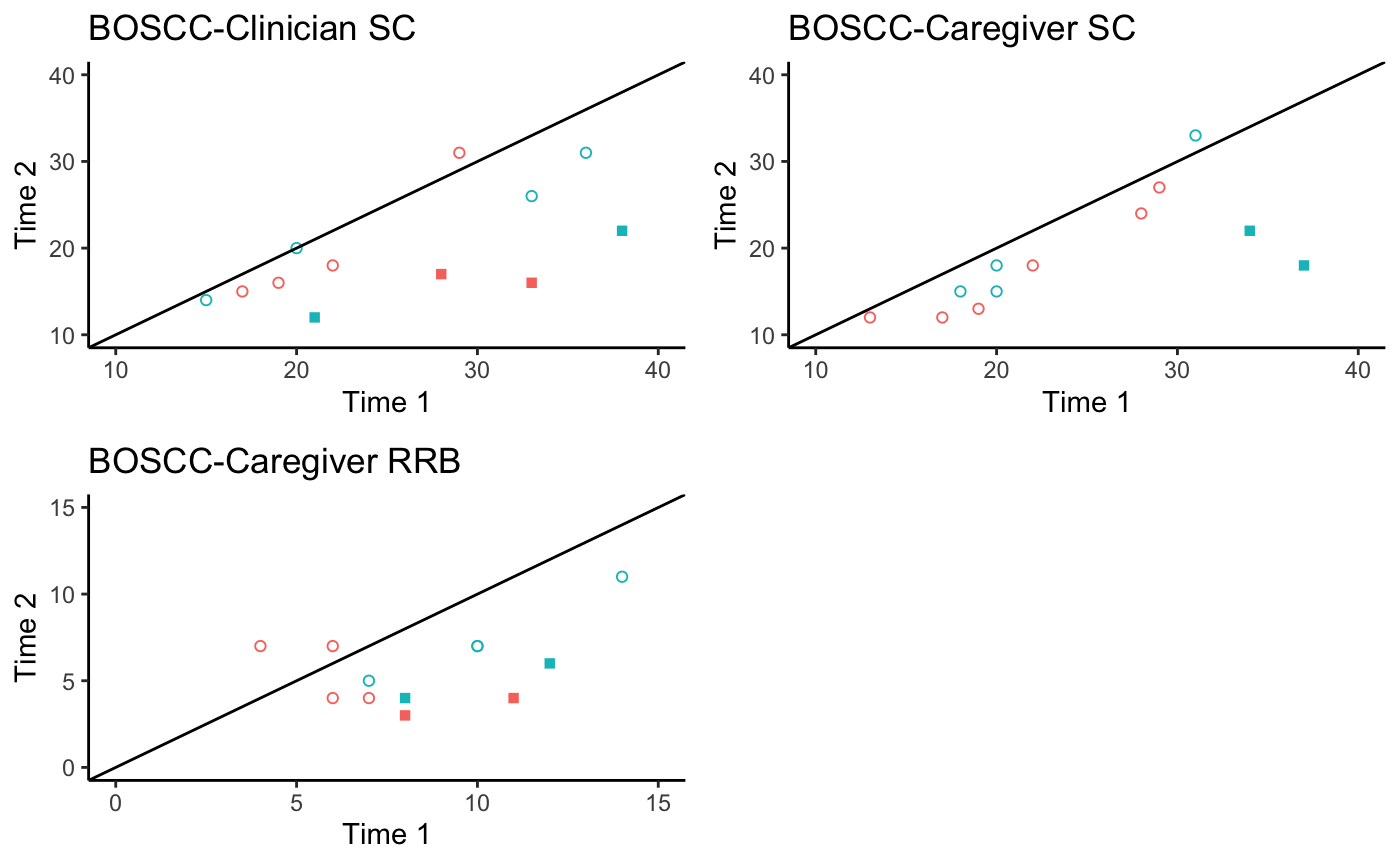

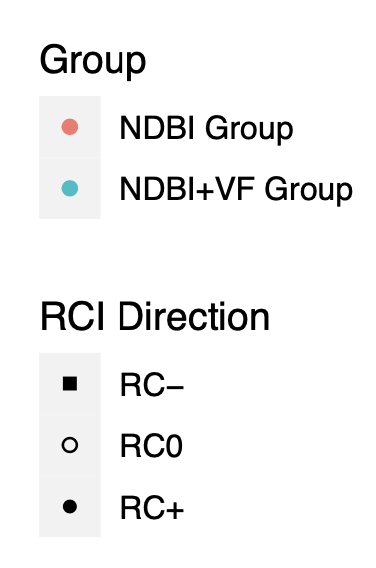


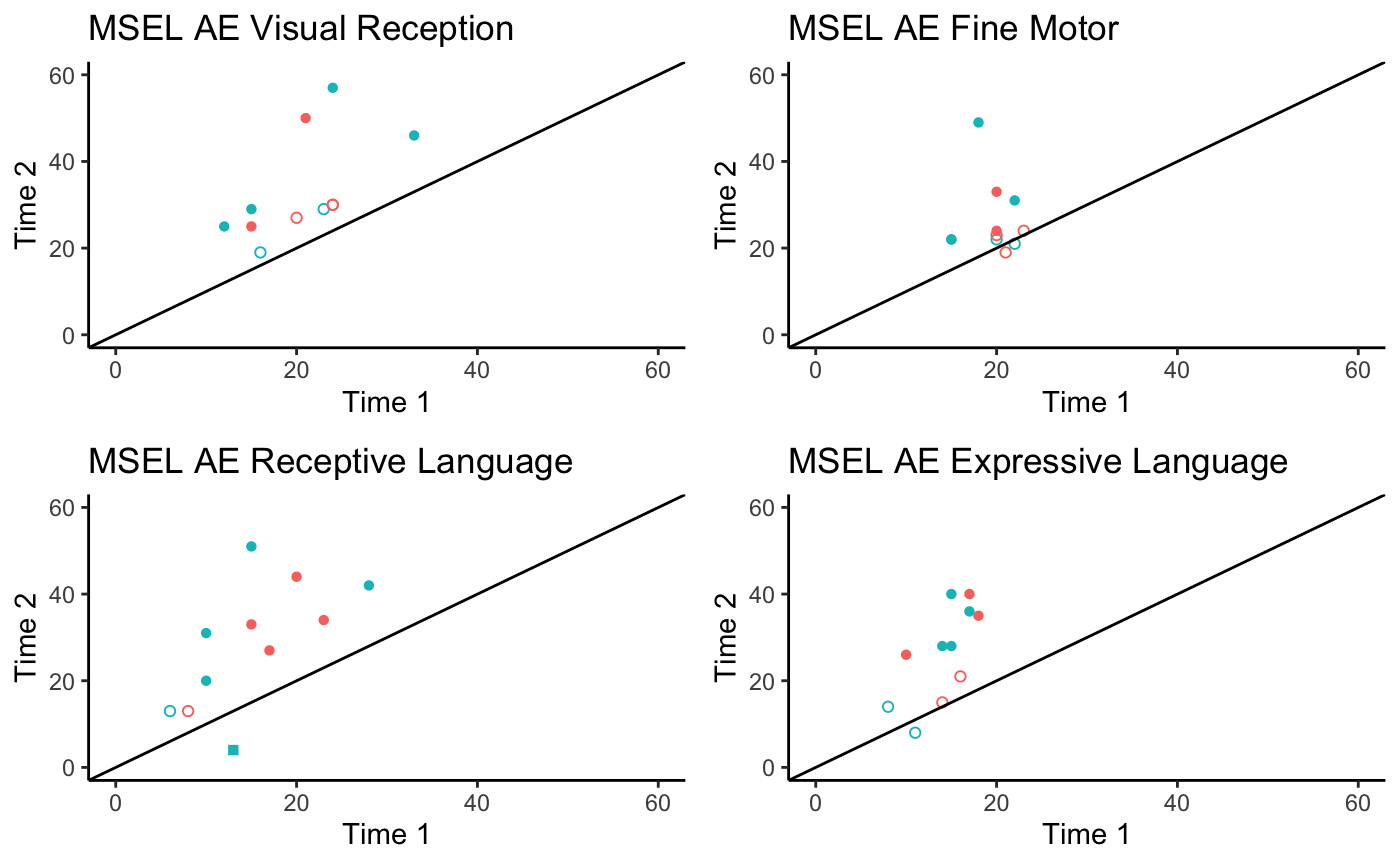


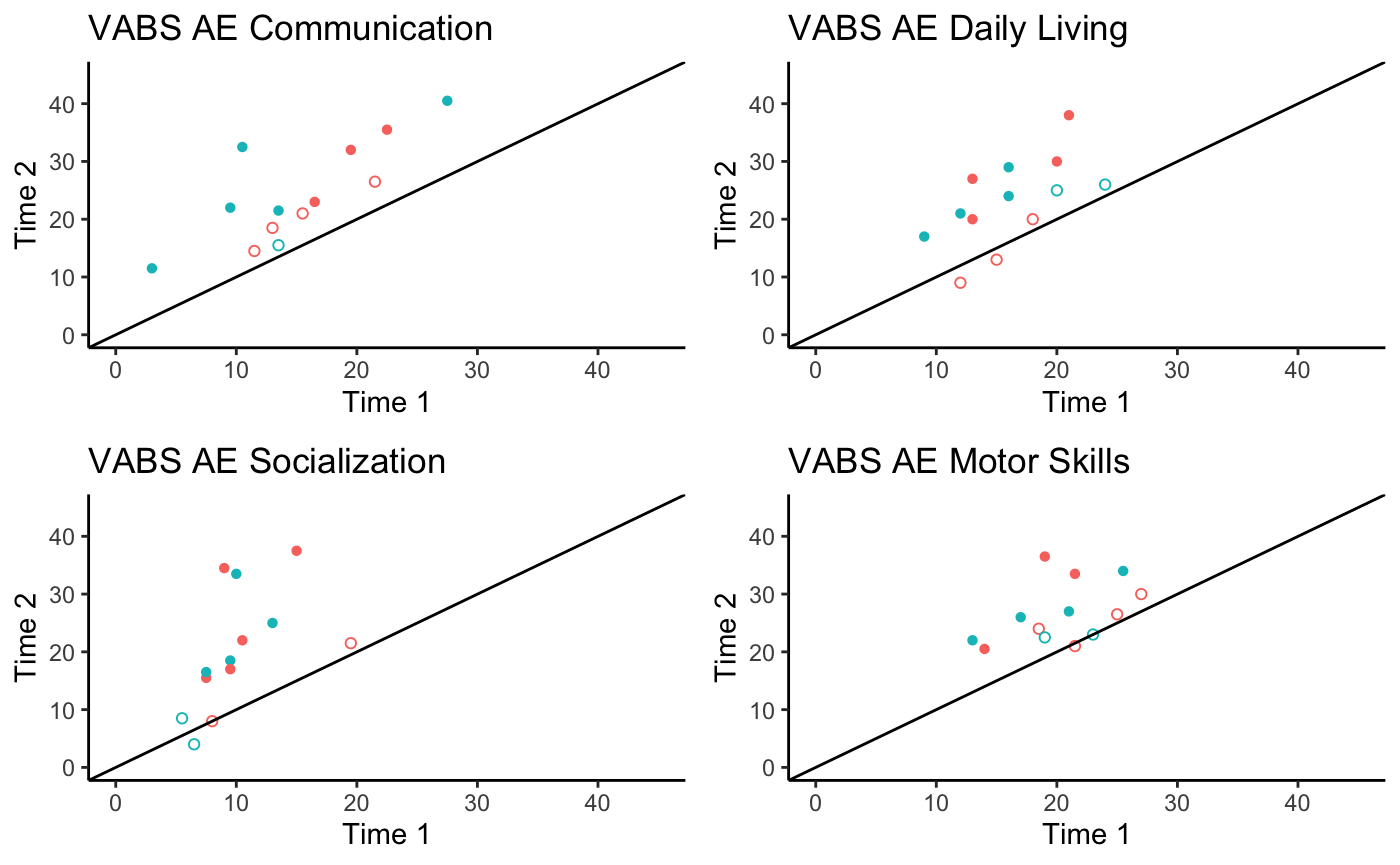

Supplement: Supplementary file 1 [file Data_Sheet_1.docx]
